# Screening of High-Yield 2-Phenylethanol Producing Strain from Wild-Type *Saccharomyces cerevisiae* and Optimization of Fermentation Parameters

**DOI:** 10.3390/foods14142444

**Published:** 2025-07-11

**Authors:** Chenshuo Zhang, Tingwen Fan, Zhichun Wang, Jiamu Yu, Xiaoming Guo, Wei Jiang, Lili Miao, Huaiyi Yang

**Affiliations:** 1 State Key Laboratory of Microbial Diversity and Innovative Utilization, Institute of Microbiology, Chinese Academy of Sciences, Beijing 100101, China; s20233020273@cau.edu.cn (C.Z.); fantw@im.ac.cn (T.F.); 13936531341@163.com (X.G.); 2 State Key Laboratory of Animal Biotech Breeding, College of Biological Sciences, China Agricultural University, Beijing 100193, China; jiangwei01@cau.edu.cn; 3 Technology Development and Transfer Center, Institute of Microbiology, Chinese Academy of Sciences, Beijing 100101, China; wangzc@im.ac.cn; 4 Department of Life Sciences, Imperial College London, South Kensington Campus, London SW7 2AZ, UK; james.yu21@imperial.ac.uk; 5 School of Life Science, Dalian Minzu University, Dalian 116600, China; 6 University of Chinese Academy of Sciences, Beijing 100049, China; 7 Beijing Key Laboratory of Genetic Element Biosourcing & Intelligent Design for Biomanufacturing, Beijing 100101, China

**Keywords:** *Saccharomyces cerevisiae*, 2-phenylethanol, strain screening, fermentation condition optimization

## Abstract

2-Phenylethanol (2-PE), an aromatic alcohol with a rose-like fragrance, is widely used in the food, pharmaceutical, and high-end cosmetic industries. In this study, a high-yield 2-PE-producing strain was isolated and identified as *Saccharomyces cerevisiae* based on morphological characterization and taxonomic identification. Fermentation medium components (carbon and nitrogen sources) were optimized through single-factor experiments in shaking flasks, and fermentation medium with 40 g/L glucose, 5 g/L malt extract, 1.75 g/L corn steep liquor, 2.5 g/L yeast extract, 5 g/L malt extract, 1.75 g/L corn steep liquor was considered suitable for 2-PE production. RT-qPCR results indicated that corn steep liquor activates expression of genes related to the shikimate pathway and Ehrlich pathway (*pha2*, *aro4*, *aro8*, and *aro9*), thereby promoting the synthesis of 2-PE through these pathways. Excess yeast extract inhibited the expression of *aro8* and *aro9*, while enhancing the expression of *tdh3* and *adh2*, thus promoting the de novo synthesis of 2-PE. Furthermore, fermentation in a 5 L bioreactor was applied to investigate the effects of feeding strategies, inoculum proportion, and pH on 2-PE production. With a pH of 5.5 and10% inoculum proportion, the supplementation of the substrate L-Phe led to a 2-PE production of 4.81 g/L after 24 h of fermentation. Finally, in situ product recovery (ISPR) techniques was applied to alleviate 2-PE cytotoxicity, achieving a production of 6.41 g/L. This process offers a promising strategy for producing 2-PE efficiently and naturally, paving the way for further industrial applications in food, pharmaceutical, and cosmetic sectors.

## 1. Introduction

2-Phenylethanol (2-PE) is an aromatic alcohol derivative that possesses a characteristic and pleasant rose-like fragrance. 2-PE is widely utilized in daily applications due to its GRAS (Generally Recognized as Safe) status [1]. This designation confirms its safety for intentional use in food and beverage applications under specified conditions. 2-PE is used as an essential ingredient in high-quality cosmetic perfumes for creating rose-type and other floral scent profiles [2,3]. The high-boiling-point of 2-PE contributes to the aromatic profile of various alcoholic beverages. It readily forms esters with acids in wine, which contribute distinctly to the pleasant fruity aroma of wine [4]. Additionally, 2-PE is commonly employed as a flavoring agent in beverages, pastries, ice cream, and chewing gum [5]. 2-PE also exhibits significant antimicrobial properties. Researchers have explored the potential of 2-PE as an antimicrobial agent for protecting fruits, food products, and flowers [6,7]. According to Zou et al., 2-PE effectively inhibits the growth of *Botrytis cinerea* both in lab cultures and on strawberry, reducing natural disease while maintaining fruit quality under room temperature storage conditions [8]. Sun et al. have reported that 2-PE interferes with protein synthesis and degradation in *Fusarium graminearum*, significantly reducing deoxynivalenol production and providing effective control against *Fusarium* head blight in wheat [9]. Given its widespread use, the global demand for 2-phenylethanol (2-PE) is approximately 10,000 tons per year [10]. Currently, the majority of 2-PE is produced via chemical synthesis, which is often environmentally unfriendly [11]. Although extraction from plants is an alternative, this process proves excessively expensive (approximately US $1000/kg) and fails to meet commercial scale demand [12,13]. Consumers become increasingly conscious of environmental and health issues and consistently ask for affordable supplies, motivating suppliers to seek biotechnological alternatives for 2-PE production [14].

**Figure 1:**
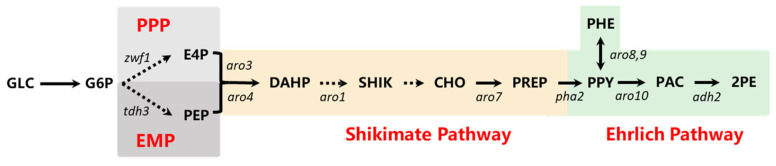
Metvbabolic pathway of 2-phenylethanol in microorganisms. GLC glucose, G6P glucose-6-phosphate, E4P erythose-4-phosphate, PEP phosphoenolpyruvate, DAHP 3-deoxy-D-arabino-heptulosonic acid 7-phosphate, SHIK shikimate, CHO chorismate, PREP prephenate, PPY phenylpyruvate, PHE phenylalanine, PAC phenylacetaldehyde, 2PE 2-phenylethonal.

Today, with the development of synthetic biology, *S. cerevisiae* has used as an ideal chassis organism for producing diverse bioproducts, including terpenes, steroids, naringenin and so on [15,16,17]. *S. cerevisiae* can produce 2-PE naturally through both shikimate pathway and Ehrlich pathway (Figure 1). The high efficiency of the Ehrlich pathway makes it an attractive option for 2-PE biosynthesis, where L-phenylalanine (L-Phe) is converted to phenylpyruvate by transaminase and then decarboxylated to form phenylacetaldehyde, which is further converted into 2-PE [18]. As the regulatory pathways of 2-PE biosynthesis became clearer, researchers developed engineered strains to boost its synthesis efficiency [19,20]. However, the use of synthetic molecules produced by metabolic engineering strains is forbidden in food sectors. Screening wild-type strains and fermentation conditions optimization are economical strategies to enhance 2-PE production efficiency. Chreptowicz et al. isolated 28 yeast strains from plant materials and fermented food samples, among which a *S. cerevisiae* JM2014 strain demonstrated the highest 2-PE titer, reaching 3.28 g/L [21]. Shu et al. demonstrated that *S. cerevisiae* achieves high cell density growth at 25 °C with an aeration rate of 1.3 vvm, whereas 2-PE biosynthesis is favored at 35 °C and 2 vvm. Implementing a two-stage fermentation strategy (switching from 25 °C to 35 °C after 72 h) increased 2-PE yield by 19% compared to single-stage fermentation [22].

Although *S. cerevisiae* can naturally produce 2-PE, the commercial viability of this process has been limited by relatively low yield. To overcome this limitation, isolating and screening yeast strains with higher 2-PE production capabilities is crucial [23]. This study aims to optimize fermentation medium and parameters for high-yielding strains to maximize 2-PE productivity, while exploring the molecular mechanisms by which key nitrogen source regulate 2-PE biosynthesis. In this study, a high-yield *S. cerevisiae* D-22 was isolated and identified. Then single-factor experiments was conducted to examine the effects of fermentation medium composition on 2-PE production. Besides, the effect of the transcript level related to key 2-PE biosynthesis genes under different nitrogen source conditions was investigated by RT-qPCR. Further scale-up culture was conducted in a 5 L bioreactor to test effect of feeding strategies, inoculum proportion and pH on 2-PE production. Finally, in situ product recovery (ISPR) was employed to alleviate cellular inhibition of 2-PE and 2-PE production reached a high level, suggesting that the *S. cerevisiae* D-22 is an efficient and economical 2-PE producer.

## 2. Materials and Methods

### 2.1. Isolation of Yeast Strains

Twenty-seven strains were screened from our laboratory preserved microbial collection. To obtain single colonies, three rounds of streaking were carried out on YPD plate. Twenty-seven single colonies were cultured in YPD medium using shake flask fermentation at 30 °C for 36 h to assess 2-PE production.

### 2.2. Scanning Electron Microscopy

A single colony was isolated from a freshly YPD plate and was inoculated in a culture of 5 mL YPD medium for 16 h at 30 °C with 220 rpm. The culture media was centrifuged at 5000 rpm for 2 min and the supernatant was discard. The cell pellet was resuspended in 2.5% (wt/vol) glutaraldehyde immediately, and the suspension was incubated overnight at 4 °C for cell fixation. Fixed structures were washed three times with ddH_2_O. After centrifugation, the samples were sequentially dehydrated in a graded ethanol series (50%, 70%, 85%, and 95% (vol/vol)), and finally three times for 15 min in absolute ethanol. They were then dried in a Leica EM CPD300 (Leica, Wetzlar, German). Subsequently, the dried materials were mounted on a conductive stub using double-sided cellulose tape and sputter-coated with gold under an argon atmosphere. Samples were observed using a Hitachi SU8010 field-emission scanning electron microscope (Hitachi, Tokyo, Japan). An accelerating voltage of 3 kV was used [24].

### 2.3. Identification and Phylogenetic Analysis of the Isolated Yeast

To identify the isolated yeast strain, 18S rRNA sequencing analysis was carried out. Yeast genomic DNA was extracted using the TIANamp Yeast DNA Kit (Tiangen Biotech Co., Ltd., Beijing, China). The 18S rRNA gene was amplified using the genomic DNA as a template, with primers listed in Table A1. PCR was performed under the following conditions: an initial denaturation at 98 °C for 5 min, followed by 35 cycles of denaturation at 95 °C for 30 s, annealing at 55 °C for 30 s, and elongation at 72 °C for 1 min. A final extension step was conducted at 72 °C for 10 min. (The enzyme was purchased from Yeasen Biotechnology Co., Ltd. Shanghai, China) Purified PCR products were sequenced by Sangon Biotech Co., Ltd. (Shanghai, China). The sequencing results were aligned using nucleotide BLAST (https://blast.ncbi.nlm.nih.gov/ 20 May 2025). Fifteen strains exhibiting high homology were selected as representative strains. *Hanseniaspora uvarum* NRRL Y-1614, a distantly related genus belonging to the same family, was included as an outgroup. Phylogenetic tree was constructed using MEGA (version 11.0, Pennsylvania State University, University Park, PA, USA) based on the neighbor-joining method with 1000 bootstrap replications [25].

### 2.4. Growth Conditions for Shake Flask Fermentation

Isolated *S. cerevisiae* D-22 cells were cultured on YPD plates at 30 °C for 2 days. A single colony was picked and placed in 50 mL fresh SM (seed medium, 40 g/L glucose, 2.5 g/L corn steep liquor, 20 g/L yeast extract, 10 g/L Malt extract, 6 g/L KH_2_PO_4_, 0.4 g/L MgSO_4_·7H_2_O, pH = 6.5, designed for rapid microbial propagation) and cultured overnight at 30 °C with shaking at 220 rpm to prepare the precultures. For shake flask fermentation, FM (fermentation medium, 40 g/L glucose, 1.75 g/L corn steep liquor, 2.5 g/L yeast extract, 5 g/L Malt extract, 6 g/L KH_2_PO_4_, 0.4 g/L MgSO_4_·7H_2_O, 0.2 g/L CaCl_2_, 8 g/L L-Phe, pH = 6.0, designed for both cell growth and 2-PE production) was modified from a previous study [26]. Precultures were inoculated at a final proportion of 10% (vol/vol) into 45 mL fermentation medium in a 500 mL shake flask and incubated at 30 °C with 220 rpm for 72 h.

### 2.5. RT-qPCR Assays for Yeast

*S. cerevisiae* D-22 cells were cultured for 24 h in three different media: fermentation medium (CK), a modified medium with elevated yeast extract concentration (increased from 2.5 g/L to 10 g/L, Yeast extract +), and another modified medium with complete removal of corn steep liquor (reduced from 1.75 g/L to 0 g/L, Corn steep liquor -). Following fermentation, cells were collected for total RNA extraction and subsequent RT-qPCR analysis to determine the transcript level related to key 2-PE biosynthesis pathway. Total RNA was extracted using RNAprep Pure Cell/Bacteria Kit (Tiangen Biotech Co., Ltd., Beijing, China) according to the manufacturer’s instructions. 1 µg RNA was treated with M5 Super qPCR RT kit with gDNA remover (Mei5 Biotechnology Co., Ltd., Beijing, China) at 42 °C for 2 min to remove DNA and at 37 °C for 15 min to reverse transcription. The cDNA was used as the template to perform qPCR with Hieff UNICON^®^ Universal Blue qPCR SYBR Green Master Mix (Yeasen Biotechnology Co., Ltd., Shanghai, China). The *β-actin* gene served as the internal control. Data were analyzed with LightCycler^®^ 4.2 Software [27]. Primers used for RT-qPCR analysis are listed in Appendix A, Table A2.

### 2.6. Fermentation in 5 L Fermenter

The *S. cerevisiae* D-22 cells were first grown in SM, and cultivated at 30 °C with 220 rpm for 24 h, then inoculated into 2 L FM in 5 L fermenter with a 10% (vol/vol) inoculum proportion (The seed culture has an OD_600_ of 19.35 ± 0.78). The 5 L fermentation was conducted under the following conditions: temperature was controlled at 30 ± 0.2 °C, aeration was set at 0.8 L/min, and dissolved oxygen (DO) was maintained at 35%. The pH was regulated at 5.5 ± 0.05 by automatic addition of 5 M NaOH. A feeding strategy was employed using 400 g/L glucose at a rate of 5–20 mL/h. The fermentation last from 24 h to 36 h.

### 2.7. Analysis Methods

Optical density was measured by using a Metash 721 spectrophotometer (Metash, Shanghai, China) at 600 nm.

The glucose concentrations in the supernatant were analyzed via the enzymatic autoanalyzer SBA-40C (Biology Institute of Shandong Academy of Sciences, Jinan, China). 25 μL of standard solution (1 g/L glucose solution) is injected into the SBA-40C. This procedure is repeated until the instrument automatically calibrates to 100. Then, the sample to be tested is injected, and the instrument displays the measured glucose concentration.

The 2-PE and L-Phe concentrations in the supernatant were analyzed via Agilent 1200 Series HPLC System (Agilent Technologies, Inc., Santa Clara, CA, USA) with a diode array detector (DAD) with a C18 column (250 × 4.6 mm, Phenomenex, Torrance, CA, USA). The mobile phase consisted of 0.1% formic acid (solvent A) and acetonitrile (solvent B), delivered at a flow rate of 0.8 mL/min. The column temperature was maintained at 25 °C, and the injection volume was 5 µL. The gradient elution program with a binary pump is shown in Table 1.

### 2.8. Statistical Analysis

Results in this study were presented as mean values with corresponding standard deviations (SD) (means ± S.D) of three independent assays. Statistical analysis of significant differences was performed using GraphPad Prism 8 (version 8.3.0.538, GraphPad Software, USA).

## 3. Results

### 3.1. Isolation of the 2-PE-Producing Strains

**Figure 2:**
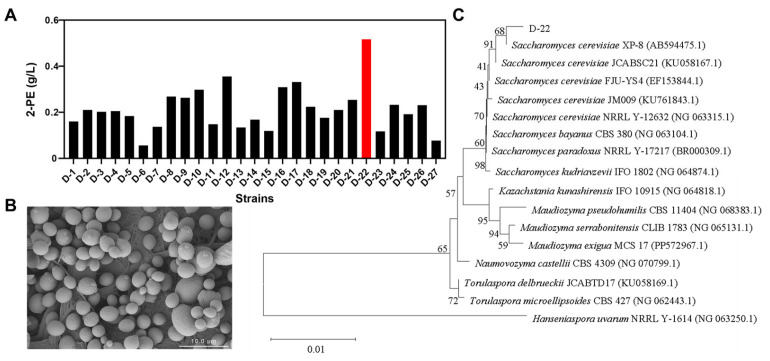
Isolation of the 2-PE-producing strains. (**A**) Comparison of 2-PE production of different strains in YPD medium; (**B**) Scanning electron microscope images of *S. cerevisiae* D-22; (**C**) Neighbor-joining phylogenetic tree based on 18S rRNA gene sequences of *S. cerevisiae* D-22.

Twenty-seven yeast strains were isolated and cultured in YPD liquid medium. After 36 h of fermentation, a faint rose fragrance could be detected from the medium, suggesting the potential production of 2-phenylethanol (2-PE). Then, the 2-PE were analyzed using high-performance liquid chromatography (HPLC). Almost all of the yeasts were capable of producing 2-PE to some extent. However, the level of 2-PE producing from one strain which was named D-22 in this study is significantly higher than other strains and it reached to 517 mg/L (Figure 2A). Thus, strain D-22 was selected for further investigation. Using scanning electron microscope to perform morphological identification of strain D-22. As evident in the SEM micrographs (Figure 2B), a population of predominantly oval to spherical cells was observed, exhibiting smooth surface morphology with diameters ranging from 2 to 10 μm. Some cells exhibited budding structures, where daughter cells emerged as surface protrusions morphologically identical to mother cells. Following bud detachment, characteristic circular scar structures (bud scars) remained on the mother cell surface, demonstrating typical features of budding yeast reproduction. 18S rRNA of strain D-22 was sequenced and aligned using NCBI blast to identify the biological classification status of strain D-22 (Figure 2C). It was found that D-22 was clustered with *S. cerevisiae* XP-8 (GenBank accession number: NG 063315.12), and the 18S rRNA gene sequence of D-22 had 99.73% homology with XP-8. Based on morphoagronomic characterization and phylogenetic analysis, strain D-22 was identified as *S. cerevisiae* and designated as *S. cerevisiae* D-22.

### 3.2. Effect of Fermentation Medium Composition on 2-PE Production

**Figure 3:**
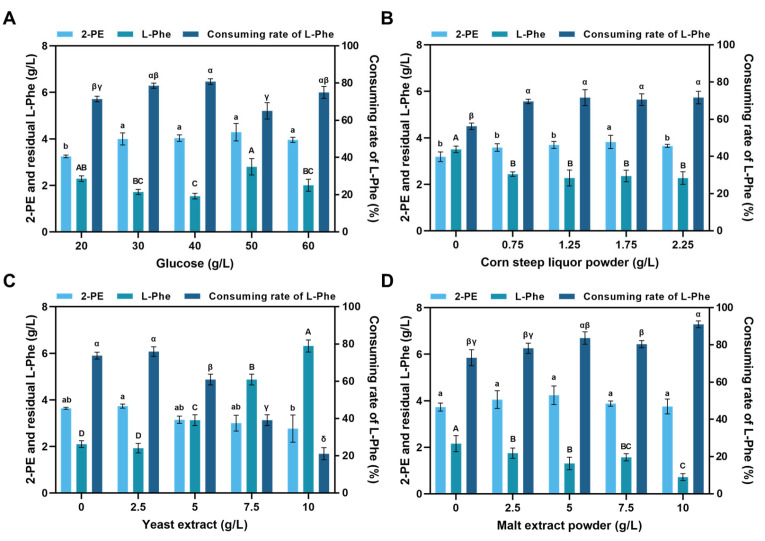
Influence of initial glucose and nitrogen sources concentration on 2-PE production. *S. cerevisiae* D-22 was cultured in FM medium with different concentrations of (**A**) initial glucose, (**B**) corn steep liquor, (**C**) yeast extract and (**D**) Malt extract for 72 h. The concentrations of both L-Phe and 2-PE were analyzed using HPLC. Error bars represent the standard deviation of three independent assays. Different letters indicated the significant difference based on one-way analysis of variance (ANOVA) (*p* < 0.05).

To further improve the 2-PE production, fermentation medium (40 g/L glucose, 1.25 g/L corn steep liquor, 5 g/L yeast extract, 5 g/L Malt extract, 6 g/L KH_2_PO_4_, 0.4 g/L MgSO_4_·7H_2_O, 0.2 g/L CaCl_2_, 8 g/L L-Phe, pH = 6.0) was employed and modified. Glucose plays a crucial role in both cell growth and product synthesis. Therefore, optimizing its initial concentration is essential for improving 2-PE production [28]. To increase 2-PE production, the optimized medium was obtained by single factor experiments. The effect of different initial glucose concentrations (20, 30, 40, 50 and 60 g/L) on 2-PE production was tested (Figure 3A). The 2-PE production was reached 4.03 g/L and 4.29 g/L with 40 g/L and 50 g/L initial glucose respectively. However, significant differences were observed in residual L-Phe concentrations (1.54 g/L and 2.80 g/L), corresponding to consumption rates of 80.80% and 65.05% respectively. The highest 2-PE production was achieved with 50 g/L initial glucose concentration, but this condition resulted in a 15.75% reduction in L-Phe consumption efficiency compared to the 40 g/L initial glucose concentration, leading to substantial substrate wastage. When the glucose concentration was higher than 50 g/L, the transformation of L-Phe decreases. Although 50 g/L glucose resulted in slightly higher 2-PE production than 40 g/L, the difference was not statistically significant (*p* > 0.05). Overall, a higher initial glucose concentration promoted 2-PE synthesis by enhancing L-Phe conversion, but considering both efficiency and resource utilization, 40 g/L glucose was selected for subsequent experiments.

The effect of three different nitrogen sources (corn steep liquor, yeast extract and malt extract) at different concentrations on 2-PE production was tested. The effect of corn steep liquor concentrations on 2-PE production was tested (Figure 3B). Interestingly, corn steep liquor as nitrogen source exhibited a positive effect on L-Phe transportation (Figure 3B). The absence of corn steep liquor significantly reduced the L-Phe transportation, resulting in a residual L-Phe concentration of 3.50 g/L. When 1.75 g/L of corn steep liquor was added to the medium, the residual L-Phe concentration decreases by approximately 32.57% compared to when it was not added. With the rise of yeast extract concentration, the residential L-Phe concentration increased, and the 2-PE production decreased (Figure 3C). These results indicate that yeast extract has a certain inhibitory effect on L-Phe transportation. However, the 2-PE production reached 2.76 g/L (0.023 mol/L) and with 10 g/L yeast extract when 1.69 g/L (0.010 mol/L) L-Phe was consumed. This suggests that approximately 0.013 mol/L (1.58 g/L) of 2-PE was synthesized from glucose, demonstrating that yeast extract can promote the strain’s de novo synthesis of 2-PE. The concentration of malt extract was positively correlated with L-Phe transportation and 2-PE production. As the concentration of malt extract increases, the residual L-Phe concentration gradually decreases. The 2-PE production reached 4.23 g/L with 5 g/L malt extract (Figure 3D). After optimized by single-factor experiments, the medium was adjusted to 40 g/L glucose, 1.75 g/L corn steep liquor, 2.5 g/L yeast extract, 5 g/L malt extract, 6 g/L KH_2_PO_4_, 0.4 g/L MgSO_4_·7H_2_O, 0.2 g/L CaCl_2_, and 8 g/L L-Phe, and the concentration of 2-PE increased from 4.03 g/L to 4.23 g/L. Additionally, the reduced yeast extract addition lowered the overall cost.

### 3.3. Effect of Different Nitrogen Source Conditions on 2-Phenylethanol Biosynthesis Genes

**Figure 4:**
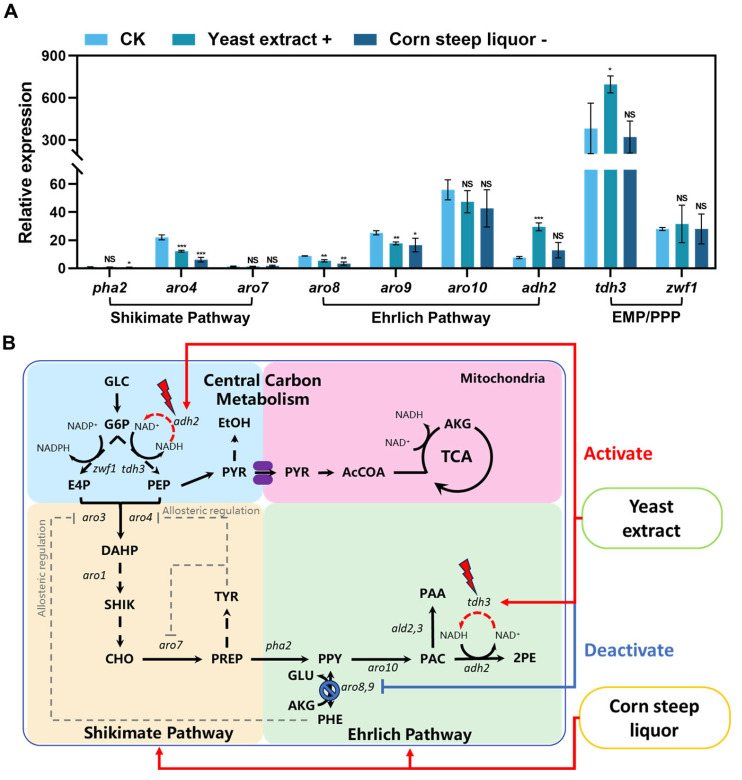
Influence of different nitrogen source conditions on 2-PE biosynthesis genes. (**A**) RT-qPCR assay to confirm the effect of the transcript level related to key 2-PE biosynthesis genes under different nitrogen source conditions. The transcript level of *pha2* in the control group (CK) was set to 1 for normalization. Error bars represent the standard deviation of three independent assays. Asterisks indicated the significant difference based on unpaired Student’s *t*-test (* *p* < 0.05, ** *p* < 0.01, *** *p* < 0.001); (**B**) GLC glucose, G6P glucose-6-phosphate, E4P erythose-4-phosphate, PEP phosphoenolpyruvate, DAHP 3-deoxy-D-arabino-heptulosonic acid 7-phosphate, SHIK shikimate, CHO chorismate, PREP prephenate, TYR L-tyrosine, PPY phenylpyruvate, PHE L-phenylalanine, PAC phenylacetaldehyde, PAA phenylacetate, 2PE 2-phenylethonal.

The results above demonstrated that yeast extract facilitates the de novo synthesis of 2-PE in *S. cerevisiae* D-22, while corn steep liquor promotes 2-PE production via the Ehrlich pathway. To investigate the effect of yeast extract and corn steep liquor on 2-PE biosynthesis genes, *S. cerevisiae* D-22 was cultured for 24 h in three different media: fermentation medium (CK), a modified medium with increased yeast extract concentration from 2.5 g/L to 10 g/L (Yeast extract +), and another modified medium with decreased corn steep liquor concentration from 1.75 g/L to 0 g/L (Corn steep liquor -). Following fermentation, cells were collected for total RNA extraction and subsequent RT-qPCR analysis to determine the transcript level related to key 2-PE biosynthesis pathway, including *aro3*, *aro4*, *aro7*, and *pha2* (shikimate pathway); *aro8*, *aro9*, *aro10*, and *adh2* (Ehrlich pathway); *tdh3* (Embedn-Meyerhof-Parnas pathway); and *zwf1* (pentose phosphate pathway) (Figure 4A).

Through three independent experimental replicates, the Ct values for *aro3* consistently exceeded 32, indicating that *aro3* remains transcriptionally silent under these culture conditions. The supplement of corn steep liquor resulted in upregulation of key genes in both the shikimate pathway and Ehrlich pathway (*pha2*, encoding prephenate dehydratase; *aro4*, encoding DAHP synthase; *aro8*, encoding aromatic aminotransferase I; and *aro9*, encoding aromatic aminotransferase II), consequently promoting both L-Phe consumption and 2-PE production. Supplement with excessive yeast extract triggered downregulation of *aro8* and *aro9* in *S. cerevisiae* D-22, impairing the conversion of L-Phe to phenylpyruvate and thereby inhibiting the metabolic flux from L-Phe to 2-PE. However, the transcript level of *tdh3* and *adh2* were significantly upregulated, leading to improve in 2-PE production from de novo synthesis. Therefore, corn steep liquor activates both shikimate pathway and Ehrlich pathway, thereby significantly increasing 2-PE production. Yeast extract inhibits conversion of L-Phe to 2-PE and enhances the de novo synthesis of 2-PE in *S. cerevisiae* D-22.

### 3.4. Effect of pH and Inoculum Proportion on 2-PE Production

**Figure 5:**
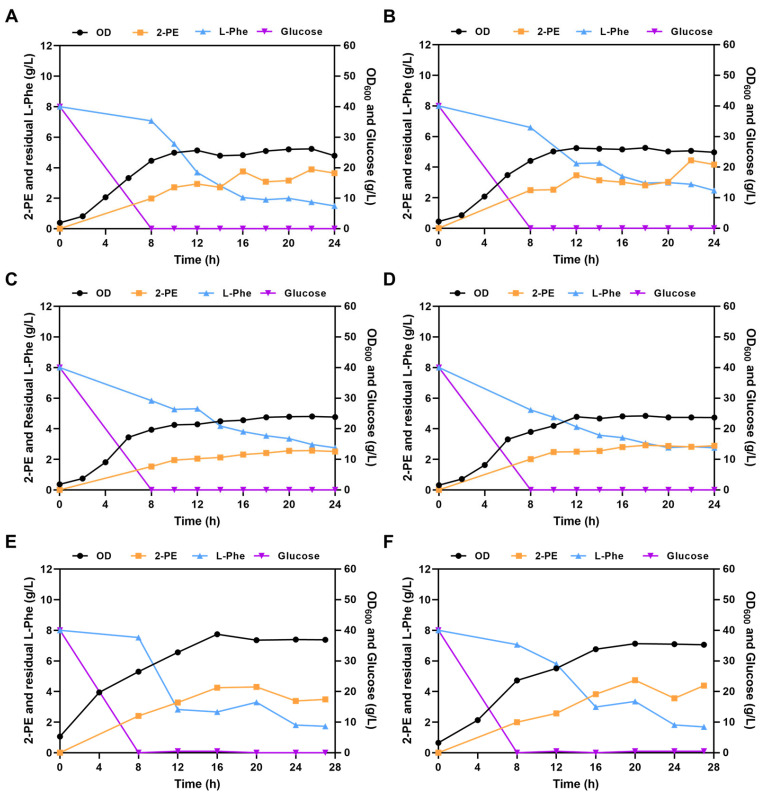
Influence of pH and inoculum proportion on 2-PE production. Cell growth, glucose and L-Phe consumption, and 2-PE production with (**A**) pH = 5; (**B**) pH = 5.5; (**C**) pH = 6; (**D**) pH = 7; (**E**) 20% inoculum proportion; (**F**) 10% inoculum proportion.

Fed-batch fermentation of *S. cerevisiae* D-22 in 5 L bioreactor was carried out to improve 2-PE production. Fermentation medium containing 40 g/L glucose, 1.75 g/L corn steep liquor, 2.5 g/L yeast extract, 5 g/L Malt extract, 6 g/L KH_2_PO_4_, 0.4 g/L MgSO_4_·7H_2_O, 0.2 g/L CaCl_2_, 8 g/L L-Phe. Since *S. cerevisiae* D-22 is a wild-type strain, its optimal growth pH remains undetermined. To evaluate the influence of pH on fermentation, experiments were conducted at constant pH values ranging from 5.0 to 7.0 with a 10% inoculum proportion. Under a pH of 5.0, the 2-PE production reached 3.89 g/L at 22 h of fermentation (Figure 5A). Under a pH of 5.5, the 2-PE production peaked at 22 h, reaching 4.44 g/L (Figure 5B). However, under a pH of 6.0 and 7.0 (Figure 5C,D), the 2-PE production significantly decreased, which highest 2-PE production reached only 2.57 g/L and 2.83 g/L, respectively. Yeast thrives in slightly acidic environment, typically with an optimal growth pH range of 5.0–6.0. As the pH exceeded 6.0, 2-PE synthesis was inhibited, indicating that fermentation pH significantly influences the production of 2-PE by *S. cerevisiae* D-22.

Inoculum proportion could influence the accumulation of target metabolites. Insufficient biomass at lower inoculum levels may slow down product synthesis, while excessive inoculation can lead to inefficient substrate utilization and reduced yields [29]. To increase the performance of *S. cerevisiae* D-22 in 2-PE production, the influence of different inoculum proportion was investigated under a pH of 5.5 (Figure 5E,F). After 16 h of fermentation, the 2-PE production with a 20% inoculum proportion reached 4.25 g/L, which is an 11% increase compared to the 10% inoculum proportion. However, after 20 h of fermentation, the 2-PE yield with a 10% inoculum proportion reached 4.74 g/L, which is a 10% increase compared to the 20% inoculum proportion.

### 3.5. Using ISPR Technology to Alleviate Inhibitory Effects and Enhance 2-PE Production

**Figure 6:**
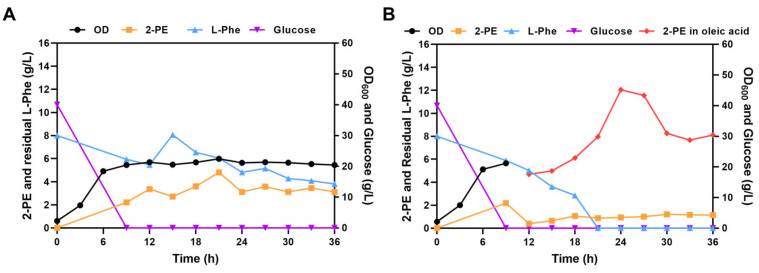
Fed-batch fermentation of *S. cerevisiae* D-22 in a 5 L bioreactor. Cell growth, glucose and L-Phe consumption, and 2-PE production. (**A**) following the addition of 2 g/L L-Phe at 12 h; (**B**) following the addition of 1 L oleci acid at 10 h. Discussion.

**Figure A1:**
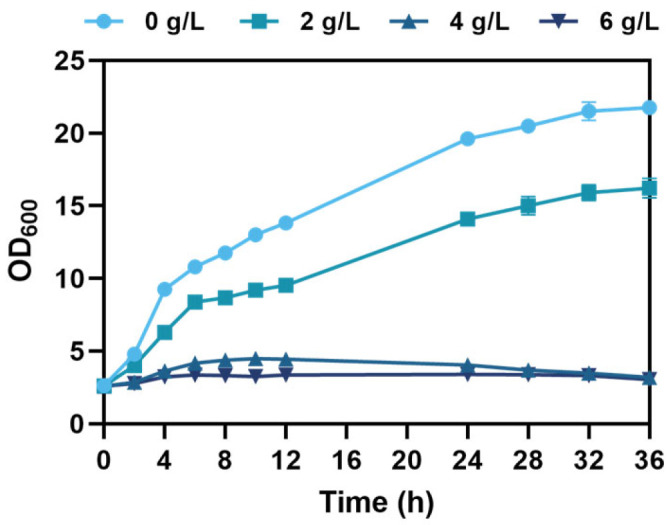
The effect of different 2-PE concentrations on cell growth of *S. cerevisiae* D-22.

In the later stages of fermentation, consumption of L-Phe decreased, possibly due to accumulation of product (2-PE), leading to chemical equilibrium and reduced conversion of L-Phe to 2-PE. To drive the metabolic flux toward 2-PE synthesis, an additional 2 g/L of L-Phe was added at 12 h. As a result, 2-PE production increased to 4.81 g/L at 21 h, with a productivity of 0.229 g/L/h (Figure 6A). This enhancement may attribute to the availability of more substrate to conversion. However, the maximum biomass decreased to 22.4 (OD_600_), which could be due to the inhibitory effect of elevated 2-PE concentrations on cell growth [10]. Therefore, the growth curve of *S. cerevisiae* D-22 was monitored in YPD medium containing different concentrations of 2-PE to assess its tolerance to 2-PE (Figure A1). The OD_600_ of the strain rapidly increased during 0–24 h (log phase), and gradually plateaued between 24–36 h (stationary phase) under 0 and 2 g/L of 2-PE concentration, indicating healthy growth of strain. However, the OD_600_ remained consistently low all the time when 2-PE concentration exceeded 4 g/L, indicating a significant cell damage.

Due to the toxicity of 2-PE, which inhibits cell growth, in situ product recovery (ISPR) technology was employed to recover 2-PE onto the extractant, alleviating the inhibitory effects of 2-PE and promoting chemical equilibrium towards 2-PE synthesis to enhance its production (Figure 6B). At 10 h of fermentation, 1 L of oleic acid was added to the medium, and the 2-PE production in the aqueous phase rapidly decreased from 2.18 g/L at 9 h to 0.396 g/L at 12 h. At 24 h, the 2-PE production in the oil phase reached 12.04 g/L, while in the aqueous phase it was 0.94 g/L, resulting in a total 2-PE production of 6.41 g/L. This represents a 33% increase compared to the fermenter supplemented with L-Phe, with a productivity of 0.267 g/L/h.

**Figure 7:**
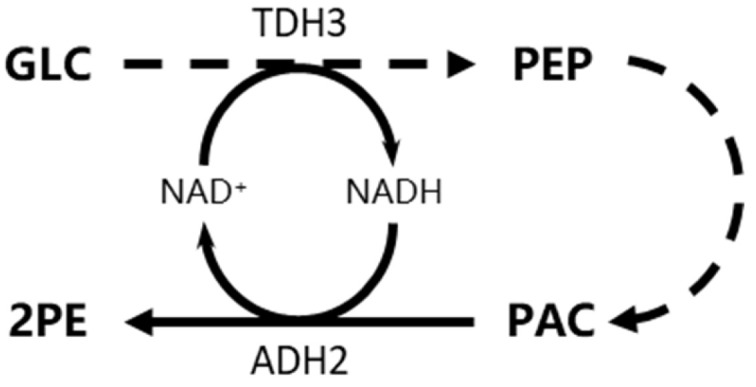
The upregulation of *adh2* and *tdh3* sustains NAD^+^/NADH homeostasis and enhances the de novo synthesis of 2-PE. GLC glucose, PEP phosphoenolpyruvate, PAC phenylacetaldehyde, 2PE 2-phenylethonal, TDH3 glyceraldehyde-3-phosphate dehydrogenase, ADH2 alcohol dehydrogenase 2.

2-Phenylethanol (2-PE) is widely used in daily life. Beyond its extensive applications in food industry, perfumes and cosmetics, its antimicrobial activity also provides a foundation for developing novel green food preservatives. Many yeasts can naturally produce 2-phenylethonal (2-PE), but low production has limited the commercial application. In our work, a wild-type strain *S. cerevisiae* D-22 that highly efficient produced of 2-PE was screened. We obtained fermentation medium suitable for production of 2-PE by optimized fermentation medium composition. Besides, we observed that 2-PE production increases while L-Phe conversion decreases with high glucose. Yeast can produce 2-PE through both shikimate pathway (using glucose as the substrate) and Ehrlich pathway (using L-Phe as the substrate). Once Ehrlich pathway was shut down by low L-Phe conversion, shikimate pathway can be used as alternative pathway to synthesis 2-PE. It is not contradiction that 2-PE production increase when L-Phe conversion decreases as Figure 4 and had demonstrated that yeast extract facilitates the de novo synthesis of 2-PE. We presume that high glucose also can simulated de novo synthesis the 2-PE production. To investigate the effect of yeast extract and corn steep liquor on 2-PE production, we detected the transcript level of 2-PE biosynthesis-related genes in different medium using RT-qPCR. *aro3* remains transcriptionally silent under these culture conditions, which may be attributed to feedback inhibition of the DAHP synthase (ARO3) by L-Phe [30]. The presence of excessive L-Phe in the medium inhibits DAHP synthase enzymatic activity and *aro3* gene expression. DAHP synthase is one of key enzymes in the shikimate pathway, catalyzing the condensation of erythrose-4-phosphate (E4P) and phosphoenolpyruvate (PEP) to form 3-deoxy-D-arabino-heptulosonate-7-phosphate (DAHP). DAHP synthase has two isoenzymes: ARO3 and ARO4, which are feedback-inhibited by L-Phe and L-Tyr, respectively. Overexpressing feedback inhibition-resistant mutants via site-directed mutagenesis is an effective strategy to alleviate amino acid-mediated suppression of DAHP synthase. This approach can enhance carbon flux toward the shikimate pathway, thereby further increasing 2-PE production [31]. Since ARO7 (chorismate mutase) and PHA2 (prephenate dehydratase) exhibit limited native expression, overexpression of *aro7* and *pha2* may increase phenylpyruvate (precursor of 2-PE) supply, potentially elevating 2-PE production. ARO8 (aromatic aminotransferase I) and ARO9 (aromatic aminotransferase II) catalyze the conversion of L-Phe to phenylpyruvate. Excessive yeast extract supplementation and removal of corn steep liquor downregulated the expression of *aro8* and *aro9*, resulting in decreased 2-PE production, consistent with previous reports [32]. In addition to insufficient precursor supply, the efficiency of biocatalysis is also constrained by inadequate cofactor supply and regeneration. The transcript level of *tdh3* (encoding glyceraldehyde-3-phosphate dehydrogenase) and *adh2* (encoding alcohol dehydrogenase 2) were significantly upregulated. Glyceraldehyde-3-phosphate dehydrogenase (TDH3) catalyzes the conversion of glyceraldehyde-3-phosphate (G3P) to 1,3-bisphosphoglycerate, subsequently generating phosphoenolpyruvate (PEP, one of a key precursor of shikimate pathway) while concomitantly reducing NAD^+^ to NADH. Subsequently, alcohol dehydrogenase 2 (ADH2) then utilizes NADH to reduce phenylacetaldehyde (PAC) to 2-PE, simultaneously regenerating NAD^+^. Therefore, the upregulation of *tdh3* enhanced PEP and NADH production, supplying both the PEP (precursor of 2-PE) and NADH (reducing equivalents) required for 2-PE synthesis. Besides, the upregulation of *adh2* channeled metabolic flux toward 2-PE biosynthesis and sustaining NAD^+^/NADH homeostasis through its redox activity. This coordinated regulation created a recyclable and efficient metabolic network that enhanced the de novo synthesis of 2-PE (Figure 7). Wang et al. established a cofactor self-sufficient system in *Escherichia coli* by coupling glutamate dehydrogenase with transaminase and alcohol dehydrogenase. This design enabled simultaneous regeneration of the cosubstrate (2-oxoglutarate) and redox cofactor (NAD(P)H), thereby enhancing 2-PE production [33]. Therefore, cofactor/redox imbalance strategy is feasible for enhanced production of diverse chemicals.

To further improve 2-PE production, fed-batch fermentation was performed in a 5 L bioreactor. The highest 2-PE production was reached at a pH of 5.5, and 2-PE production significantly decreased as the pH exceeded 6.0. It showed that pH has a significant impact on the 2-PE production. The highest biomass (OD_600_) reached 26.18, 26.30, 23.12 and 24.20 under a pH of 5.0, 5.5, 6.0 and 7.0. Therefore, pH influences cellular growth and thus affects the accumulation of production. Yeast prefers slightly acidic conditions, while its growth is inhibited under neutral conditions, thereby limiting product synthesis. Besides, the pH of the medium has a significant effect on the ionic state of the substrate and affect the adsorption capacity of 2-PE on the adsorbent [12]. The isoelectric point (pI) of L-Phe is 5.48. Maintaining the fermentation pH close the value of pI may enhance its uptake and subsequent conversion to 2-PE by yeast cells, potentially improving 2-PE production. In summary, controlling the pH within an optimal range in the fermentation process can enhance cell growth and 2-PE production. A higher 2-PE production was observed with a 10% inoculum proportion than a 20% inoculum proportion. This could be due to the morphological alterations in cells induced by high cell density, which in turn restrict the biosynthesis of aromatic alcohols [34]. Concurrently, a higher inoculum proportion could introduce substantial metabolic byproducts, inhibiting both cell growth and fermentation. Furthermore, accelerated nutrient depletion during early growth phase leads to insufficient nutrient for the later stages of fermentation.

In the later stages of fermentation, consumption of L-Phe decreased, possibly due to accumulation of product (2-PE), leading to chemical equilibrium and reduced conversion of L-Phe to 2-PE. To shift the equilibrium toward 2-PE synthesis, additional substrate (L-Phe) was supplemented during fermentation, resulting in a maximum 2-PE yield of 4.81 g/L. However, the more decline in L-Phe was not accompanied with more 2-PE production, and the maximum biomass only reached 22.4 (OD_600_). Tolerance test of *S*. *cerevisiae* D-22 to 2-PE revealed severe inhibition at a concentration of 4 g/L. Thus, the limited biomass and production bottleneck may be attributed to the toxicity of high 2-PE concentrations. To alleviate the inhibitory effect of 2-PE on the cell, in situ product recovery (ISPR) techniques was implemented to maintain low 2-PE concentration in the medium. 2-PE exhibits antimicrobial activity, and it primarily breaks the cell membrane [35]. As a monounsaturated fatty acid, oleic acid can modulate membrane fluidity and maintain its structural integrity, thereby stabilizing cellular homeostasis. Therefore, oleic acid was selected as the ISPR extractant for dual purposes: alleviating 2-PE cytotoxicity by reducing its concentration in the medium, and driving the equilibrium toward enhanced 2-PE synthesis. Preliminary experiments indicated that 2-PE was primarily produced during the logarithmic phase, and its excessive accumulation causes the strain to enter the stationary phase more rapidly. Therefore, oleic acid was introduced as an extractant before 2-PE concentration reached inhibitory level to remove 2-PE from the medium. The 2-PE concentration in the medium rapidly decreased and remained low level after oleic acid was added. Additionally, no L-Phe was detected in the medium after 21 h, and the 2-PE content in the oleic acid phase peaked at 24 h. This suggests that reducing 2-PE concentration alleviated cellular inhibition and enhanced the conversion of L-Phe to 2-PE. Thus, significantly enhanced both the strain’s viability and 2-PE productivity.

## 4. Conclusions

In this study, a wild-type strain of *S*. *cerevisiae* D-22 that naturally produces high levels of 2-phenylethonal (2-PE) has been identified. The fermentation conditions were optimized by adjusting the initial glucose and nitrogen concentrations in the fermentation medium. Analysis of transcript level of 2-PE biosynthesis related genes under different nitrogen source conditions revealed that yeast extract enhanced de novo synthesis of 2-PE, and corn steep liquor enhanced both shikimate pathway and Ehrlich pathway and promoted 2-PE production in *S. cerevisiae* D-22. Furthermore, controlling pH, and inoculum proportion were optimized to enhance 2-PE production in 5 L bioreactor. Under a pH of 5.5 and 10% inoculum proportion, in situ product recovery (ISPR) techniques was employed to recover 2-PE, alleviating the inhibitory effects of 2-PE. As a result, a 2-PE production of 6.41 g/L with a productivity of 0.267 g/L/h was achieved in a 5 L bioreactor. The results indicated that *S. cerevisiae* D-22 was a competitive strain for 2-PE production.

## Figures and Tables

**Table 1:** Gradient elution procedure of mobile phase.

Time (min)	Mobile Phases A (%)	Mobile Phases B (%)
0	99	1
7	99	1
15	70	30
30	0	100
35	0	100
35.1	99	1
42	99	1

## Data Availability

The original contributions presented in this study are included in the article. Further inquiries can be directed to the corresponding author.

## Abbreviations

The following abbreviations are used in this manuscript:

2-PE	2-Phenylethonal
AKG	α-Ketoglutaric acid
CHO	Chorismate
DAHP	3-Deoxy-D-arabino-heptulosonic acid 7-phosphate
E4P	Erythrose-4-phosphate
EMP	Embden–Meyerhof–Parnas pathway
FDA	Food and drug administration
GLU	Glutamate
GRAS	Generally recognized as safe
ISPR	In situ product recovery
Phe	Phenylalanine
PAA	Phenylacetate
PABA	*Para*-Aminobenzoic acid
PAC	Phenylacetaldehyde
PEP	Phosphoenolpyruvate
PPP	Pentose phosphate pathway
PPY	Phenylpyruvate
PREP	Prephenate
S3P	Shikimate-3-phosphate
SHIK	Shikimate
TYR	Tyrosine

## Appendix A

**Table A1:** List of primers used in this study.

Primers	Sequence (5′–3′)	Description
NS1	GTAGTCATATGCTTGTCTC	Amplification of 18S rRNA fragment for sequencing
NS8	TCCGCAGGTTCACCTACGGA	
qP-actin-F	GCCCCAGAAGCTTTGTTCCA	Amplification of *β-actin* for RT-qPCR
qP-actin-R	GATGGAGCCAAAGCGGTGAT	
qP-aro3-F	TTGTTTCTCCTTGGGTGCCA	Amplification of *aro3* for RT-qPCR
qP-aro3-R	TTCAGTGCCCACGATAGCAG	
qP-aro3-F2	ACTGTTGGCTGGAAAGGGTT	Amplification of *aro3* for RT-qPCR
qP-aro3-R2	TGGCACCCAAGGGAAACAA	
qP-aro4-F	CGTCGGCTGGAAAGGTCTAA	Amplification of *aro4* for RT-qPCR
qP-aro4-R	GAGATTCGGTGGTTCTGGCA	
qP-aro7-F	ACTTCGGTTCTGTTGCCACT	Amplification of *aro7* for RT-qPCR
qP-aro7-R	CTCGTTGGTAGGGTCCACAC	
qP-pha2-F	GCCAGCAAGACTTTGAGGGT	Amplification of *pha2* for RT-qPCR
qP-pha2-R	GCTGCTGGGAGGTACTCAAC	
qP-aro8-F	TCACCCAAGCCTCCTTTTCC	Amplification of *aro8* for RT-qPCR
qP-aro8-R	TGGCTAAAACGTCCCAGTCC	
qP-aro9-F	ATGTCATCCTTTCTGGCGGG	Amplification of *aro9* for RT-qPCR
qP-aro9-R	TGGGACTCTCTGTCGAAGGT	
qP-aro10-F	GATTTCGCGTTTCCTTCGCA	Amplification of *aro10* for RT-qPCR
qP-aro10-R	CTGCACCGTCACCTTCAAAC	
qP-adh2-F	CGCAGTCGTTAAGGCTACCA	Amplification of *adh2* for RT-qPCR
qP-adh2-R	CCCACGTAAGAGCCGACAAT	
qP-zwf1-F	TCCCGCCTTATTTGGGCTTT	Amplification of *zwf1* for RT-qPCR
qP-zwf1-R	CGACGTTGGCACTTTTCTCG	
qP-tdh3-F	ACTGTCCACTCTTTGACTGCT	Amplification of *tdh3* for RT-qPCR
qP-tdh3-R	CGACGGTTGGGACTCTGAAA	

**Table A2:** Ct values of RT-qPCR.

	*β-actin*	*pha2*	*aro4*	*aro7*	*aro8*	*aro9*	*aro10*	*adh2*	*tdh3*	*zwf1*	*aro3*
CK	21.41	26.54	21.99	26.15	23.28	21.76	20.40	23.52	19.58	ND	31.35
	21.71	26.45	22.02	26.21	23.28	21.82	20.74	23.57	18.18	20.89	32.20
	21.22	26.21	21.81	25.67	23.24	21.65	20.67	23.32	18.06	20.96	32.29
Yeast extract +	21.53	26.41	22.66	25.95	23.98	22.12	20.91	21.37	17.32	21.38	33.42
	20.98	26.34	22.53	25.84	23.55	22.00	20.49	21.14	17.28	20.44	ND
	21.15	26.26	22.52	25.56	23.79	21.96	20.46	21.36	17.09	21.45	33.42
Corn steep liquor -	20.76	27.07	23.81	25.57	24.66	22.36	20.98	22.53	17.86	20.69	ND
	21.91	28.14	24.59	26.40	25.68	23.25	21.92	23.06	18.96	21.87	ND
	21.02	27.33	24.04	26.19	24.79	22.59	21.20	23.83	18.09	20.96	ND

ND: Not detected.
